# Polyphenol Content and Modulatory Activities of Some Tropical Dietary Plant Extracts on the Oxidant Activities of Neutrophils and Myeloperoxidase

**DOI:** 10.3390/ijms13010628

**Published:** 2012-01-09

**Authors:** Cesar N. Tsumbu, Ginette Deby-Dupont, Monique Tits, Luc Angenot, Michel Frederich, Stephane Kohnen, Ange Mouithys-Mickalad, Didier Serteyn, Thierry Franck

**Affiliations:** 1 Interfacultary Centre of Drug Research (CIRM), Laboratory of Pharmacognosy, Department of Pharmacy, Faculty of Medicine, Hospital Avenue 1, B36, University of Liège, Sart Tilman, 4000 Liège 1, Belgium; E-Mails: M.Tits@ulg.ac.be (M.T.); L.Angenot@ulg.ac.be (L.A.); M.Frederich@ulg.ac.be (M.F.); 2 Centre for Oxygen Research and Development (CORD), Institute of Chemistry B6a, University of Liège, Sart Tilman, 4000 Liège 1, Belgium; E-Mails: cord@ulg.ac.be (G.D.-D.); stephane.kohnen@celabor.be (S.K.); amouithys@ulg.ac.be (A.M.-M.); didier.serteyn@ulg.ac.be (D.S.); 3 Department of Clinical Sciences, Large Animal Surgery, Faculty of Veterinary Medicine, B41, University of Liège, Sart Tilman, 4000 Liège 1, Belgium

**Keywords:** neutrophils, myeloperoxidase, reactive oxygen species, green vegetables, polyphenols, antioxidant

## Abstract

Young leaves *of Manihot esculenta* Crantz (Euphorbiaceae), *Abelmoschus esculentus* (Malvaceae), *Hibiscus acetosella* (Malvaceae) and *Pteridium aquilinum* (Dennstaedtiaceae) are currently consumed as green vegetables by peoples in sub-Saharan Africa, Latin America, Asia and their migrants living in Western Europe. Sub-Saharan peoples use *Manihot*, *Abelmoschus* and *Hibiscus* also in the folk medicine to alleviate fever and pain, in the treatment of conjunctivitis, rheumatism, hemorrhoid, abscesses, ... The present study investigates the effects of aqueous extracts of those plants on the production of reactive oxygen species (ROS) and the release of myeloperoxidase (MPO) by equine neutrophils activated with phorbol 12-myristate 13-acetate (PMA). The ROS production was measured by lucigenin-enhanced chemiluminescence (CL), and the release of total MPO by an ELISA method. The study also investigates the effect of the extracts on the activity of MPO by studying its nitration activity on tyrosine and by using a new technique called SIEFED (**S**pecific **I**mmunological **E**xtraction **F**ollowed by **E**nzymatic **D**etection) that allows studying the direct interaction of compounds with the enzyme. In all experiments, the aqueous extracts of the plants developed concentration-dependent inhibitory effects. A moderate heat treatment did not significantly modify the inhibitory capacity of the extracts in comparison to not heated ones. Total polyphenol and flavonoid contents were determined with an HPLC-UV/DAD analysis and a spectroscopic method using Folin-Ciocalteu reagent. Some polyphenols with well-known antioxidant activities (caffeic acid, chlorogenic acid, hyperoside, rosmarinic acid and rutin) were found in the extracts and may partly explain the inhibitory activities observed. The role of those dietary and medicinal plants in the treatment of ROS-dependent inflammatory diseases could have new considerations for health.

## 1. Introduction

Neutrophils ingest infectious agents by phagocytosis and kill them by proteolysis and reactive nitrogen and oxygen species (RNOS/ROS) produced by oxidant enzymes such as myeloperoxidase (MPO) and others. When neutrophils undergo excessive activation, MPO may be released in the extracellular milieu [1]. In stimulated neutrophils, NADPH-oxidase (NOx) produces superoxide anion (O_2_^•−^), which dismutates into hydrogen peroxide (H_2_O_2_). This reactive compound is a substrate for MPO to produce hypochlorous acid (HOCl), a strong oxidant compound at the origin of the highly reactive and fugacious hydroxyl radical (^•^OH) [2–4]. Inducible NOsynthase (iNOs) produces nitric oxide (^•^NO), which reacts with O_2_^•−^ and HOCl to form reactive species (peroxynitrite, NO_2_Cl) or is transformed into NO_2_^−^, another substrate for MPO [2]. During MPO activity, the ferric haem is oxidized into an Fe^IV^-oxoferryl π cation radical compound (Cp I), which is reduced back into an oxoferryl not-radical compound (Cp II) and further into ferric MPO. These two mono-electronic reduction steps need electron donors (tyrosine, ascorbate, urate, catecholamines, *etc.*). When NO_2_^−^ is the electron donor for the first step, it is oxidized into NO_2_^•^ [2,3] responsible for nitrations, especially for tyrosine [5,6].

NOx, MPO and iNOs are thus specialized in a cascade production of RNOS, normally essential for host defense, enzyme activation, signal transduction, gene expression, vascular tone regulation, *etc*. [7–9]. However, excessive RNOS are deleterious for cells and tissues, since they attack biomolecules, and change their structure and function. Chronic excessive RNOS production by neutrophils is involved in the onset of a lot of diseases, including atherosclerosis [3,7,8,10,11]. Therefore, the modulation of the RNOS production appears useful to treat or prevent inflammatory diseases. In this perspective, decreasing the activity of oxidant enzymes in neutrophils is nowadays considered as a major therapeutic target [12–14]. Plant polyphenols have got growing interest in this field, and are reported to prevent or delay the onset of inflammatory diseases through antioxidant (AOX) and anti-radical properties [15,16], by acting on pathways of enzyme activation, gene expression, signal transduction, *etc*. [17–21].

Polyphenols are present in *Abelmoschus esculentus* (Malvaceae), *Hibiscus acetosella* (Malvaceae), *Manihot esculenta* Crantz (Eupohorbiaceae) and *Pteridium aquilinum* (Dennstaedtiaceae). The young leaves (and crosses) of these plants are commonly consumed as green vegetables by peoples in Western and Central Africa and by their migrants living in Western Europe. *Manihot* is also consumed in Latin America, the Philippines, Indonesia, Malaysia and other Asian countries [22–24]. In folk medicine, *Abelmoschus*, *Hibiscus* and *Manihot* leaves and seeds are used to alleviate fever, headache, rheumatism, hemorrhoid, to treat ringworms, tumor, conjunctivitis, sores and abscesses [24–26]. *Hibiscus’* leaves, flowers and calyces are used in heart and nerve conditions, as diuretic, sedative, anti-scorbutic, colorectal and intestinal antiseptic [25]. In biochemical studies, *Manihot* and *Abelmoschus’* polyphenols were shown to decompose ABTS radical, to scavenge DPPH, hydroxyl and superoxide radicals, to inhibit LDL oxidation, to chelate cupric ions and to reduce ferric ions [27–32]. Those of *Hibiscus* developed similar [25,32] and chemo-preventive activities, inhibited xanthine-oxidase and lipid peroxidation [26]. Polyphenols of *Pteridium* were also studied [33,34], however, their AOX capacity was not investigated [32].

In this perspective, we started to investigate the AOX, anti-radical activities and polyphenol content of the extracts of those four plants, and demonstrated their ability to inhibit lipid peroxidation and the formation of transient free radicals [35]. Here, we investigate their anti-inflammatory activities on *ex vivo* isolated and stimulated equine neutrophils and on MPO. The RNOS production by neutrophils was measured by lucigenin-enhanced chemiluminescence (CL) and their release of MPO by an ELISA method. The nitration and peroxidase activities of MPO were studied by a tyrosine nitration assay and SIEFED (**S**pecific **I**mmunological **E**xtraction **F**ollowed by **E**nzymatic **D**etection), an original method for the study of the direct interaction between MPO and a molecule or an extract, without interference of the reaction milieu. Boiled samples as well as not heated ones were tested in parallel to assess the effect of heat. The contents of total polyphenol and tannins were determined in the plant powders by Folin-Ciocalteu reaction, flavonoids and phenolic acids by HPLC-UV/DAD.

## 2. Results and Discussion

### 2.1. Results

#### 2.1.1. Biochemical Investigations

For the biochemical experiments, we have used aqueous extracts, because the dietary plants that we have tested are traditionally cooked in water.

##### 2.1.1.1. Cell viability in Presence of the Plant Extracts Tested

The cell viability as checked by the Trypan blue exclusion test [36] was ≥96% for the phosphate buffered saline (PBS) control and the plant extracts at concentrations from 0.1 to 10 μg/mL, indicating no cytotoxicity towards neutrophils.

##### 2.1.1.2. Effects on the Total ROS Amount Produced by Neutrophils Activated with PMA (CL assay)

**Figure 1:**
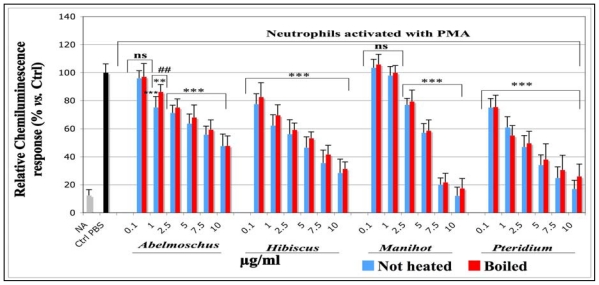
Effects of not heated and boiled aqueous extracts of *Abelmoschus esculentus*, *Hibiscus acetosella*, *Manihot esculenta* and *Pteridium aquilinum* on the lucigenin-enhanced chemiluminescence (CL) response produced by equine neutrophils stimulated with phorbol 12-myristate 13-acetate (PMA). The plant extracts were dissolved in phosphate buffered saline (PBS). The neutrophils (10^6^/well) were incubated for 10 min with the solutions of plant extracts tested at the concentrations of 0.1, 1, 2.5, 5, 7.5 and 10 μg/mL. After 10 min incubation, 25 μL CaCl_2_ (10 μM), 2 μL lucigenin (5 μM), and just before CL measurement, 10 μL PMA (16 μM) were added to the cell suspensions. For not activated (NA) cells, 10 μL of a 1% DMSO solution were added to the cell suspension instead of PMA. *p*-values (* *p* < 0.05, ** *p* < 0.01, *** *p* < 0.001) calculated by one-way ANOVA followed by Student-Newman-Keuls Multiple Comparisons Test indicated a significant effect *vs.* PBS control set as 100% response (Mean ± SD, *n* = 9). ns = not significant *vs*. PBS control. The statistical significance was also evaluated between not heated and boiled extracts and indicated as ## (*p* < 0.01).

The effects on the total ROS produced by equine neutrophils stimulated with PMA were studied with a lucigenin-enhanced CL assay. The results are shown in Figure 1.

Compared to the stimulated control cells, in which an equivalent volume of PBS was used instead of plant extracts (Ctrl PBS), the CL response decreased in a dose dependent manner for all plant extracts. A very significant inhibition (*p* < 0.001) was observed for all concentrations tested, (from 0.1 to 10 μg/mL) for *Hibiscus* and *Pteridium* and from 2.5 μg/mL for *Abelmoschus* and *Manihot*. IC_50_ and R^2^ corresponding to each sample are shown in Table 1. According to the IC_50_ values, the ROS scavenging efficacy of the extracts decreased in the following order: *Pteridium* = *Hibiscus* > *Manihot* > *Abelmoschus*. The IC_50_ were slightly higher for boiled samples than for not heated ones, but no significant differences were observed between boiled and not heated extracts, except for *Abelmoschus* at 1 μg/mL.

##### 2.1.1.3. Effects on Total MPO Amount Released by Neutrophils Stimulated with PMA (MPO-ELISA)

**Figure 2:**
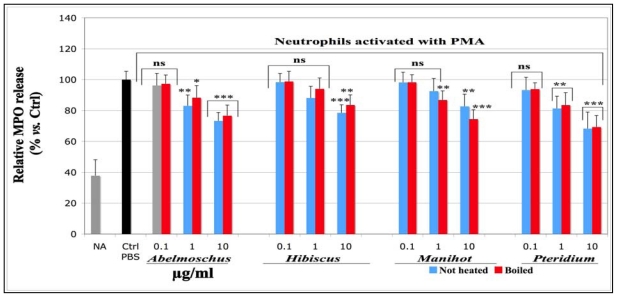
Effects of not heated and boiled aqueous extracts of *Abelmoschus esculentus*, *Hibiscus acetosella*, *Manihot esculenta* and *Pteridium aquilinum* on total myeloperoxidase (MPO) released through degranulation into the extra-cellular milieu by equine neutrophils stimulated with PMA. The aqueous extracts were solubilized in PBS. The neutrophil suspensions (10^6^/well) were incubated for 10 min with solutions of the plant extracts tested at the concentrations 0.1, 1, and 10 μg/mL and then activated for 30 min with PMA at the final concentration of 0.8 μM. After centrifugation for 10 min (450 *g*), total MPO released by the neutrophils was measured in the supernatant by an MPO-ELISA assay. *p*-values (* *p* < 0.05, ** *p* < 0.01, *** *p* < 0.001) calculated by one-way ANOVA followed by Student-Newman-Keuls Multiple Comparisons Test indicated a significant effect of the extracts *vs.* PBS control set as 100% response (Mean ± SD, *n* = 9). For not activated (NA) cells, 10 μL of a 1% DMSO solution were added to the cell suspension instead of PMA. ns = not significant *vs.* PBS control.

The total MPO released by equine neutrophils stimulated with PMA was measured by an MPO-ELISA assay developed by Franck *et al*. (2005) [37]. The findings are presented in Figure 2.

The MPO amount released by activated control cells (Ctrl PBS), in which PBS was used instead of the plant extracts, was set as 100% MPO release (Ctrl-PBS). Compared to not activated (NA) cells, neutrophils stimulated with PMA showed an important increase of total MPO released in the extra-cellular milieu. The addition of plant extracts to the final concentration of 0.1, 1 and 10 μg/mL slightly decreased the MPO release in a concentration-dependent manner. No significant effects were observed for any plant extract at the concentration of 0.1 μg/mL. The best significance (*p* < 0.001) was observed for *Abelmoschus* and *Pteridium* at 10 μg/mL. No statistical differences were observed between boiled and not heated extracts. The IC_50_ values to this assay were not determined, as the assay was done on not more than three concentration points.

##### 2.1.1.4. Effects on the Nitration Activity of MPO Measured by Tyrosine nitration

The nitration of tyrosyl residues by the peroxidasic cycle of MPO is explained by the reduction of Cp I - MPO into Cp II - MPO with the concomitant mono-electronic oxidation of nitrite (NO_2_^−^) into nitrogen dioxide (^•^NO_2_), responsible for tyrosine nitration [5]. This MPO activity was studied with UV-visible spectroscopy; the experiments were carried out at pH 5.5, the optimal pH to reach the highest yield of this enzyme activity [5,6].

**Figure 3:**
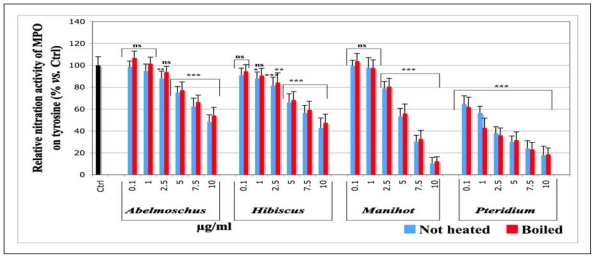
Effects of not heated and boiled aqueous extracts of *Abelmoschus esculentus*, *Hibiscus acetosella*, *Manihot esculenta* and *Pteridium aquilinum* on the nitration activity of myeloperoxidase on tyrosine. The experiments were carried out in a 100 mM acetate buffer using tyrosine (1.5 mM), MPO (0.75 μg/mL, *i.e.*, 150 mU/mL), NaCl (150 mM), H_2_O_2_ (1 mM) and NaNO_2_ (5mM). The plant extracts were dissolved in ethanol at final concentrations of 0.1, 1, 2.5, 5, 7.5 and 10 μg/mL. The control was done with an aqueous solution 1% ethanol instead of the extracts tested (Ctrl, 100% nitration activity). The reaction was performed for 30 min at 37 °C; the formation of 3-nitrotyrosine was monitored by UV-visible spectroscopy at 405 nm after alkalinization with 100 μL 0.1 M NaOH. *p*-values (* *p* < 0.05, ** *p* < 0.01, ***p < 0.001) calculated by one-way ANOVA followed by Student-Newman-Keuls Multiple Comparisons Test indicated a significant effect of the extracts *vs.* the 1% ethanol control (Ctrl) set as 100% response (Mean ± SD, *n* = 9). ns = not significant *vs*. control (Ctrl).

Compared to the control (Ctrl, black histogram), in which an equivalent volume of aqueous ethanol solution (1%) was used instead of plant extracts, and which was set as 100% nitration activity, all extracts exerted a dose dependent inhibition on the nitration activity of MPO (Figure 3). A very significant inhibition (*p* < 0.001) was observed for all concentrations tested (from 0.1 to 10 μg/mL) for *Pteridium.* The IC_50_ were slightly higher for the boiled samples than for the not heated ones, although no statistical differences were observed, and indicate that the inhibitory power decreases in the following order: *Pteridium* > *Manihot* > *Hibiscus* = *Abelmoschus*. IC_50_ and R^2^ to each sample are presented in Table 1.

##### 2.1.1.5. Effects on the Nitration-Peroxidasic Activity of MPO Measured by SIEFED Technique

The SIEFED technique is a licensed method developed by Franck *et al*. (2006) [38] for the specific detection of equine MPO and consisting on three steps. The first step is the extraction of MPO from a solution or a biological sample by its capture on specific immobilized antibodies (specific immunoextraction step). The other compounds of the sample (proteins, modulating and interfering substances) are eliminated in a following washing step. The third step is the detection of the nitration-peroxidasic activity of MPO using H_2_O_2_, nitrite anions and the fluorogenic substrate Amplex^®^ Red in the revelation solution. This experiment investigates the back reduction of the intermediate oxoferryl form (Cp II) of MPO into the ferric form using Amplex^®^ Red as electron donor, whereas NO_2_^−^ is used for the first reduction of Cp I into Cp II to enhance the amount of Cp II available for the reaction with Amplex^®^ Red.

**Figure 4:**
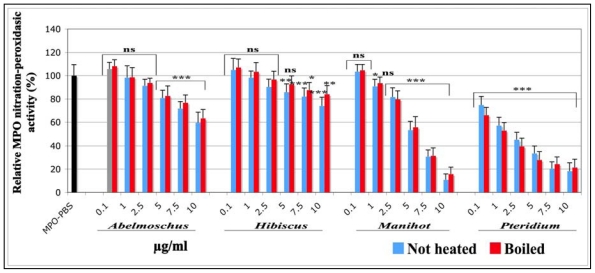
Effect of not heated and boiled aqueous extracts of *Abelmoschus esculentus*, *Hibiscus acetosella*, *Manihot esculenta* and *Pteridium aquilinum* on the MPO nitration-peroxidasic activity measured by SIEFED. The aqueous extracts were dissolved in PBS at the final concentrations of 0.1, 1, 2.5, 5, 7.5 and 10 μg/mL and incubated for 10 min with pure MPO (50 ng/mL) before performing SIEFED. *p*-values (* *p*< 0.05, ** *p* < 0.01, *** *p* < 0.001) calculated by one-way ANOVA followed by Student-Newman-Keuls Multiple Comparisons Test indicated a significant effect of the extracts *vs.* the MPO-PBS control set as 100% response (Mean ± SD, *n* = 9). ns = not significant *vs*. MPO-PBS control.

The findings to this assay are presented on Figure 4. Compared to the control (MPO PBS; black histogram), in which an equivalent volume of PBS was used instead of plant extracts, all plant extracts exerted a dose dependent inhibition on this MPO activity.

A very significant inhibition (*p* < 0.001) was observed for all concentrations tested (from 0.1 to 10 μg/mL) for *Pteridium*. The IC_50_ values indicate that the inhibitory power decreases in the following order: *Pteridium* > *Manihot* > *Abelmoschus* > *Hibiscus*. No statistical differences were observed between boiled and not heated extracts. The IC_50_ and the R^2^ corresponding to each sample are presented in Table 1.

#### 2.1.2. Phytochemical Analysis

The findings issued from the phytochemical analysis are presented on Table 2. We presented some aspects of this analysis in an earlier report [35]. Here, total polyphenols were determined with Folin-Ciocalteu reagent, flavonoids and phenolic acids were calculated by HPLC-UV/DAD analysis. In the Folin-Ciocalteu method, *Pteridium* had the highest total polyphenol, but 73% of this content were tannins. *Abelmoschus* and *Hibiscus* had respectively 41 and 29% tannins in the total polyphenol content. However, the polyphenol content of *Manihot* could not be determined. The order of total not tannin polyphenol, expressed as pyrogallol weight-equivalent in 100 g dry plant powder, was: *Pteridium* (1330 mg/100 g) > *Hibiscus* (1230 mg/100 g) > *Abelmoschus* (880 mg/100 g) (not determined for *Manihot*). In the HPLC-UV/DAD analysis, the total flavonoid content expressed as hyperoside weight-equivalent/100 g was found to decrease in the following order: *Manihot* > *Hibiscus* > *Pteridium* > *Abelmoschus*. Both *Abelmoschus* and *Pteridium* contained nearly the same amount of flavonoids. Total phenolic acid expressed as weight-equivalent of chlorogenic acid/100 g showed the following hierarchy: *Hibiscus* > *Pteridium (not* determined for *Abelmoschus* and *Manihot)*.

**Figure 5:**
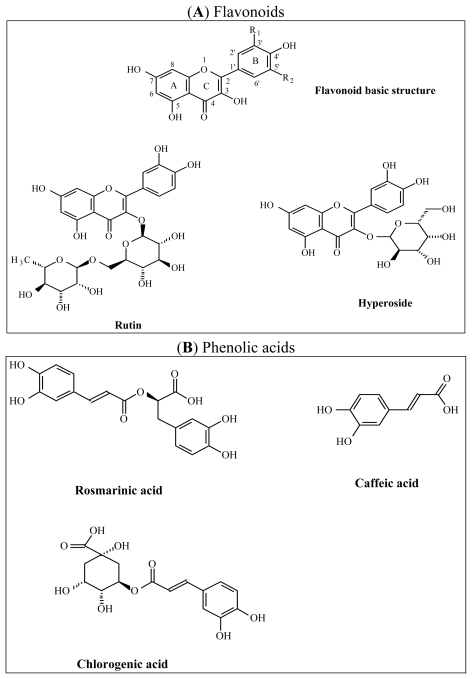
Chemical structure of flavonoids (**A**) and phenolic acids (**B**) identified in the extracts.

**Figure 6:**
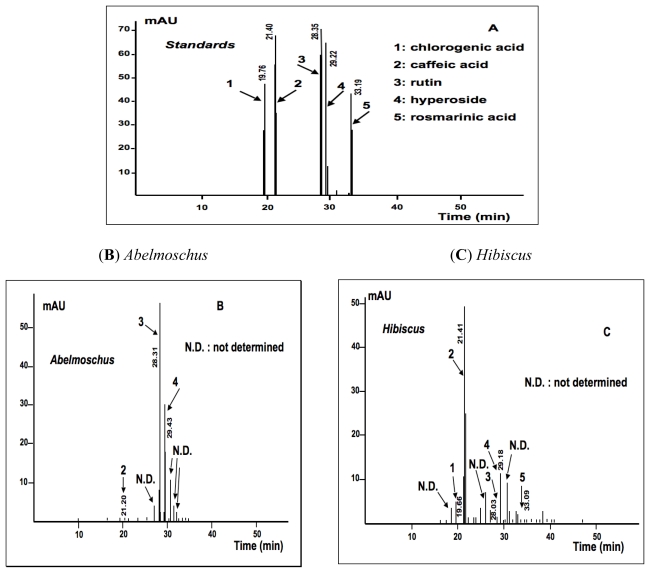

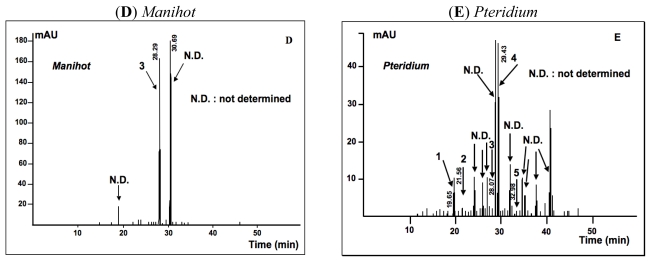
HPLC-UV/DAD chromatograms of standards and plants tested (340 nm). The chromatograms were obtained from methanolic extracts of the dry plant powders with a RP HPLC-UV/DAD analysis on a ODS column. The retention time is given in minute (horizontal axis). On the y-axis, the height of the elution peaks is expressed in arbitrary milli-unities (mAU), and the surface under these peaks, corresponds to the concentration (data not given) of the eluted compounds in the sample analyzed. (**A**) standards; (**B**) *Abelmoschus*; (**C**) *Hibiscus*; (**D**) *Manihot*; (**E**) *Pteridium*.

In the HPLC-UV/DAD analysis, some elution peaks corresponding to phenolic acids and flavonoids were identified by comparing the substances to standard purified molecules (Figures 5 and 6). In *Abelmoschus*, rutin and hyperoside were present in sufficient amount, but only few caffeic acid was detected (Figure 6B). In *Hibiscus* and *Pteridium*, the HPLC-UV/DAD analysis showed caffeic acid, chlorogenic acid and rosmarinic acid, rutin and hyperoside. *Hibiscus* contained more caffeic acid (Figure 6C) and *Pteridium* contained more hyperoside (Figure 6E). *Manihot* had a very high content of rutin and a second main compound, which is not yet determined (Figure 6D).

### 2.2. Discussion

In neutrophils and other defense cells, the RNOS production starts with the generation of superoxide anion (O_2_^•−^) by NADPH oxidase, whose activation is mediated by protein kinase C (PKC) [39]. However, an excessive RNOS production induces negative side effects for cell and health [2]. Several studies show that the inhibition of the oxidant response of neutrophils becomes a therapeutic challenge to control or modulate an excessive inflammatory response. Beside both steroidal and non-steroidal anti-inflammatory drugs (NSAIDs), there is an increasing interest to find naturally occurring agents with very few side effects for therapeutic substitution [26,40,41].

Thus, we were interested to investigate on isolated neutrophils the anti-inflammatory capacities of aqueous extracts made from some tropical dietary plants which are also used in the folk medicine to alleviate fever and pain and in the treatment of some inflammatory diseases. Here we compared the effect of the different extracts on the oxidant activity of external RNOS and on total MPO release by stimulated equine neutrophils, as well as on the nitration and peroxidasic activity of MPO.

To investigate the effect on the RNOS amount produced by neutrophils, we designed a lucigenin-enhanced chemiluminescence assay (CL) because lucigenin is a good luminescent probe to measure the O_2_^•−^ amount produced by activated neutrophils (or other inflammatory cells). In fact, lucigenin reacts specifically with O_2_^•−^ in the extra-cellular medium [42] and is divided into two symmetric luminescent adducts [43]. All extracts exerted a dose dependent inhibitory effect on RNOS amount produced by neutrophils stimulated with PMA. The inhibition effect was more powerful for *Pteridium* and *Hibiscus.* Interestingly, these two extracts contained the highest total polyphenol, all phenolic acids and flavonoids screened during the HPLC-UV/DAD analysis. The higher content of polyphenols in these extracts in comparison to *Manihot* and *Abelmoschus* can explain the more efficient inhibitory effect on ROS production.

Since the extracts were always present during the neutrophil stimulation, polyphenols can act on several ways, either by scavenging and neutralizing the RNOS produced by the cells on a direct stoichiometric relationship, or by interfering with molecules that act on the enzymes responsible for the RNOS production. Some polyphenols have been reported to interfere with the signal transduction involved in the NOx activation [17,20,21,44]. Da Cunha *et al*. (2004) demonstrated that caffeic acid, present in high concentration in *Pteridium*, exerts *in vitro* and *in vivo* anti-inflammatory actions, at least by scavenging ^•^NO and modulating the gene expression for iNOs and probably other inflammatory mediators [45]. Recently, Kim *et al*. (2011) demonstrated that hyperoside, found in high concentration in *Hibiscus*, might decrease inflammation by suppressing the activation of nuclear factor-κB in mouse peritoneal macrophages [46]. However, it appears evident that the protective effects of the extracts against the oxidant response of neutrophils could not be attributed to only one or few polyphenols but rather to a synergic effect of all polyphenols present in the extract.

Our results are in agreement with findings reported about the antioxidant activities developed by extracts of the same plants in other assays such as DPPH, ABTS, hydroxyl radical scavenging, and metal chelating [24–32]. Recently we demonstrated the potential of the extracts to decrease the oxidative response of human monocytes (HL-60 cells) activated with PMA and to inhibit lipid peroxidation [35].

Except RNOS production, neutrophils are also specialized in the release of MPO, elastase and other proteolytic enzymes outside their granules and the cell [47]. MPO is a very oxidant enzyme considered as an important marker of inflammation and cardiovascular risk [48]. Our results demonstrate that all extracts slightly decrease the MPO amount released by the neutrophils stimulated with PMA. The mechanisms involved in the degranulation of neutrophils depend on several factors such as the ligation of selectin and integrin, the phosphorylation and activation of proteins like phospholipase C and D, the intracellular Ca^2+^ uptake, the actin cytoskeleton and the activation of protein kinase C (PKC) [1,39,49,50]. Many of these factors are intracellular; it could be possible that the aqueous extracts that we tested contained not enough lipophilic compounds that could interact with the cell membrane or reach the cytosol. This moderate effect of the extracts could also be explained by the use of the stimulating agent. PMA acts directly on PKC activity and bypasses all previous events involved in the stimulation cascade of neutrophils and where some polyphenols could play a role. Interestingly, rutin was found in all extracts. Sheloum *et al*. (2003) [51] demonstrated that rutin developed anti-inflammatory activities: it interfered into the chemotaxis of neutrophils, inhibited partially the exocytosis of elastase and probably modulated the activation pathways through an interaction with PI3-kinase and mitogen-activated protein kinase (MAPK). Vauzour *et al.* (2010) [20] illustrated that flavonoids interact with MAP-kinase and PI3-kinase mediated cellular signaling and thereby induce the activation of transcription factors involved in gene expression.

Although the extracts are not very effective on the intracellular mechanisms involved in neutrophil degranulation, they could act efficiently on the activity of MPO released in the extracellular milieu. Evidence has emerged that oxidant compounds derived from the activity of MPO are necessary for their killer function toward bacterial and other pathogen agents, but also provoke tissues damages, initiate and propagate acute and chronic inflammatory diseases [8–11]. Thus, there is a growing interest to modulate the activity of oxidant enzymes with new reversible inhibitors including natural compounds [12–14,26,40,41]. We tested the effects of the extracts on the nitration and peroxidase activitiy of MPO with two techniques. All extracts exert a dose dependent inhibitory effect on both activities as measured by tyrosine nitration assay and SIEFED technique, suggesting that polyphenols interfere on the enzymatic activity of MPO. Whatever the technique used, the best effects were observed for *Pteridium* which contains the highest amount of total polyphenols and tannins, and *Manihot*, which has a high content of flavonoids. Through their chemical structure, polyphenols are efficient electron donors [15,16]. Thus, the inhibition observed on the nitration activity of MPO can be due to a competitive effect between the polyphenols of the plant extracts and NO_2_^−^ as electron donors for the reduction of Cp I - MPO into Cp II - MPO. The oxidant potential of Cp I - MPO is higher than that of Cp II - MPO. By favoring the reduction of Cp I and the accumulation of Cp II, some inhibitors have the ability to shift MPO from the chlorination cycle to the peroxidase cycle [52]. Additionally, polyphenols can also interact with NO_2_^−^, making it less available as substrate for the activity of Cp I. With SIEFED technique, we clearly demonstrate that the polyphenols present in the extracts interact directly with the enzyme [38]. Indeed, after incubation of purified MPO with the extracts, the SIEFED technique allows to capture MPO by antibodies and to wash the excess of the extracts before the enzymatic measurement of its nitration–peroxidasic activity. Any inhibition of the enzyme activity, persisting after the elimination of the extract, evidences that some molecules of the extract directly interact with the enzyme, modify its structure, hinder its active site and thus limit or block the access of substrates to this site. Several studies demonstrated the ability of purified polyphenols (resveratrol, curcumin, gallic acid, catechin) to interfere in MPO activity, to modulate [53] or to inhibit it [6,53–57].

Each plant that we have studied contains at least one polyphenol with expected AOX, anti-radical and anti-inflammatory activities resulting from its chemical structure. As seen above, the hierarchy of content was different depending on the phytochemical subclass that was considered. The inhibitory effects developed by each extract are probably the effect of a synergy resulting from all its water-soluble antioxidant compounds present in the sample, since the experiments were done with crude aqueous extracts. The HPLC-UV/DAD analysis identified clearly the presence of phenolic acids (caffeic acid, chlorogenic acid and rosmarinic acid) and flavonoids (rutin and hyperoside). The hydroxyl groups around the aromatic rings at the positions 5, 7, 3′, 4′ for hyperoside and 5, 7, 4′, 5′ for rutin, the C2-C3 double bond in the central C ring and the oxo-function on C4 are classic criteria for a powerful AOX activity by flavonoids [15,16]. Thus, these structures can sufficiently explain the inhibitory effects that we observed. However, some eluted compounds remained still unidentified and need further analysis to achieve their identification.

Usually, the plant parts that we have tested are previously chopped, immersed in water, roasted for 10 min, crushed and then cooked in water for 30 to 45 min before their consumption as green vegetables. On this way, cyanosids and ptaquilosids, well-known toxic compounds of *Manihot* and *Pteridium*, are eliminated or decrease under a not-toxic level. Consequently, the consumption provokes neither digestive problems nor allergic symptoms [58,59]. In our conditions, the not-heated samples and the boiled ones had no toxic effect on neutrophils, because either the toxic compounds were completely eliminated during the sample preparation, or the low final concentrations applied on the cells could develop no effect on cell viability. The results obtained with our model of cell viability are in agreement with this traditional use and literature data [58,59].

Moreover, the moderate heat treatment of the samples did not modify their AOX and anti-inflammatory capacities. The effects of heat treatment on the AOX potential of polyphenols found in vegetal foodstuffs depend on the matrix of the foodstuff, the kind of the bonds between the AOX compounds and other components within the matrix, as well as on the level and duration of the heat treatment. The literature reports controversial effects of heat treatment on the AOX capacity depending on the plant that was analyzed and the heat treatment that was applied [60–62]. The findings issued from our model encourage cooking green vegetables with moderate heat treatment, because such processing might preserve the AOX potential of polyphenols contained in those plants. At the same concentration, the little differences of AOX activity among the plants that we tested might be explained by the profile of polyphenols contained in each plant, and the synergy of their chemical action [15,16].

## 3. Experimental Section

### 3.1. Chemicals and Reagents

Analytical-grad methanol and ethanol, CaCl_2_, KCl, NaCl, H_2_O_2_ 30%, NaOH and Tween 20 were supplied by Merck (VWRI, Leuven, Belgium). NaNO_2_, paranitrophenyl phosphate, bovine serum albumin (BSA), ethylene diamine tetra-acetic acid (EDTA) disodium salt, *bis-N-*methylacridinium nitrate (lucigenin), phorbol 12-myristate 13-acetate (PMA), Percoll, Folin-Ciocalteu reagent, sheepskin powder, dimethylsulfoxide (DMSO), hyperoside and pyrogallol were purchased from Sigma (Bornem, Belgium). Trypan blue was from ICN Biomedicals, Inc (Ohio, USA), all aqueous solutions were prepared with water previously purified in a Milli-Q water system (Millipore, Bedford, MA, USA).

### 3.2. Vegetal Material

*Abelmoschus*, *Hibiscus*, *Manihot* young leaves and *Pteridium* crosses and leaves were gathered in Kisantu, province Bas-Congo, Democratic Republic Congo (DRC) in March 2007. The National Institute for Research in Agronomics (INRA, University of Kinshasa, DRC) authenticated the crude vegetal material and provided voucher specimens. The plant parts were dried in the dark, ground and sieved at 180 μm particle size. The recovered powder was loaded into hermetic and opaque flasks and stored at room temperature out of light until further preparations.

### 3.3. Preparation of the Plant Extracts for Biochemical Assays on Cell and Enzyme Models

For this preparation, we applied the procedure presented in our previous report [35]. Hundred g powder were stirred at 300 rpm for 6 h at room temperature in 500 mL ultra pure water until filtration and lyophilization (24 h). The lyophilized extracts were weighed and kept in hermetic and opaque flasks at −22 °C. The extraction yields increased respectively in the following order: *Abelmoschus* 6.9% < *Hibiscus* 7.3% < *Manihot* 7.4% < *Pteridium* 7.9%. For each extract, not heated and boiled samples were prepared to the final concentrations required for the assays. All preparations were carried out in darkness.

### 3.4. Isolation of Equine Neutrophils

Neutrophils were isolated from horse blood using EDTA disodium salt (1.6 mg/mL) as anticoagulant. The blood was drawn from the jugular vein of healthy horses bred and fed under identical conditions and submitted to no medical treatment (Faculty of Veterinary Medicine, University of Liège, Belgium). The neutrophils were isolated at room temperature (18–22 °C) by centrifugation (400 *g*; 45 min; 20 °C) on a discontinuous Percoll density gradient according to the method described by Pycock *et al.* (1987) [63]. The cells were gently collected and washed with two volumes of physiological saline solution. The cell pellets were resuspended in 20 mM phosphate buffered saline (PBS) at pH 7.4 with 137 mM NaCl and 2.7 mM KCl. Each batch of neutrophils was prepared with 90 mL of blood from one horse. The cells were used within 4 h after isolation. For extracts, each concentration was tested in triplicate; each assay was repeated at last three times with different batches of cells collected from different horses.

### 3.5. Preparation of the Neutrophil Activator and the Plant Extract Solution

PMA was dissolved in DMSO and aliquots were kept at −20 °C. Before use, ultra pure water was added to the aliquots to obtain a solution of 16 μM PMA concentration with 1% DMSO. Parallel assays were carried out with cell suspensions in PBS alone or PBS in 0.05% DMSO, *i.e.*, without activator of the cells. The plant extracts were solubilized in ultra pure water to the final concentrations designed respectively for each assay.

### 3.6. Biochemical Investigations

#### 3.6.1. Cell Viability Assay

The cell viability was investigated by the Trypan blue exclusion test [36] on not activated neutrophils (10^6^ cell/mL PBS) previously incubated for 1 h with the plant extracts at final concentrations of 0.1, 1, 2.5, 5, 7.5 and 10 μg/mL.

#### 3.6.2. Measurement of the Total ROS Produced by Neutrophils Activated with PMA- (CL Assay)

The ROS produced by activated neutrophils were measured by lucigenin-enhanced CL under adaptation of the method described by Benbarek *et al*. (1996) [64]. Neutrophil suspensions (10^6^ neutrophils/161 μL PBS) were distributed in the wells (10^6^ neutrophils/well) of a 96-well-microtiter plate (White Combiplate 8, Fisher Scientific) and incubated for 10 min at 37 °C in darkness with PBS-solutions of the plant extracts at final concentrations of 0.1, 1, 2.5, 5, 7.5 and 10 μg/mL. After incubation, 25 μL CaCl_2_ (10 μM) and 2 μL lucigenin (5 μM) were added into the wells. Then, the suspensions were activated with 10 μL PMA (16 μM) just before CL measurement. The CL response of the neutrophils was monitored for 30 min at 37 °C with a Fluroscan Ascent spectrophotometer (Fisher Scientific, Tournai, Belgium) and expressed as the integral value of the total CL emission. The control was performed with neutrophils activated with PMA in presence of PBS instead of the plant extracts and was taken as 100% CL response to compare with the effect developed by the plant extracts.

#### 3.6.3. Measurement of Total MPO Released in the Extra-Cellular Milieu by Neutrophils Activated with PMA (MPO-ELISA Assay)

The neutrophil suspensions (10^6^ cells/mL) were incubated for 10 min at 37 °C in darkness with PBS solutions of the plant extracts and then activated for 30 min at 37 °C once again in darkness with PMA at the final concentration of 0.8 μM. After activation, the cell suspensions were centrifuged for 10 min (450 *g*) and the supernatants were collected. To measure the total MPO released by activated neutrophils in the extra-cellular milieu, an original Equine MPO ELISA assay designed by Franck *et al*. (2005) [37] was performed, using a specific kit provided by BiopTis (Liège, Belgium). Briefly, polyclonal antibodies against equine MPO were obtained in rabbit and coated on 96 wells-microtiter plates. The supernatants, which contained MPO released by the cells, were diluted 200-fold with PBS, loaded into the wells and incubated at 4 °C overnight. After washing, a second polyclonal antibody against equine MPO, raised in guinea pigs and labeled with alkaline phosphatase, was loaded into the wells and incubated for 2 h in darkness. After an ultimate washing, the wells were loaded with a solution of paranitrophenyl phosphate for the measurement of phosphatase activity, and incubated for 30 min at 37 °C in darkness. The absorbance (405 nm) proportional to the content of MPO in the wells was read with Multiscan Ascent (Thermo Scientific). The control was performed with neutrophils activated with PMA in presence of PBS instead of the plant extracts and was taken as 100% MPO release to compare with the effects of the plant extracts.

#### 3.6.4. Measurement of the Nitration Activity of Myeloperoxidase in a Tyrosine Nitration Assay

The enzyme was purified according to the method described by Franck *et al*. 2005 [37]. The experiments were carried out in a 100 mM acetate buffer at pH 5.5 with tyrosine (1.5 mM), equine MPO (0.75 μg/mL, *i.e.*, 150 mU/mL), NaCl (150 mM), H_2_O_2_ (1 mM) and NaNO_2_ (5mM), following the method applied by Kohnen *et al.* (2007) [6]. The assays were performed in the presence of the plant extracts at the final concentrations of 0.1, 1, 2.5, 5, 7.5 and 10 μg/mL. The control was done with an aqueous solution containing 1% ethanol instead of the extracts tested. The reaction was performed for 30 min at 37 °C; the formation of 3-nitrotyrosine was monitored by UV-visible spectroscopy at 405 nm (Multiscan Ascent, Thermo Labsystem, Helsinki, Finland) after alkalinization with 100 μL of 0.1 M NaOH.

#### 3.6.5. Measurement of the Nitration-Peroxidasic Activity of Myeloperoxidase by SIEFED Technique

The MPO solution was prepared with purified equine MPO diluted in the dilution buffer (PBS 20 mM at pH 7.4 with 5 g/L BSA and 0.1% Tween-20). PBS solutions of the plant extracts to the final concentrations of 0.1, 1, 2.5, 5, 7.5 and 10 μg/mL were incubated for 10 min with MPO at a final concentration of 25 ng/mL. After incubation, the mixtures were loaded into the wells of a SIEFED microtiter plate coated with rabbit polyclonal antibodies (3 μg/mL) against equine MPO and incubated for 2 h at 37 °C in darkness. After washing up the wells, the activity of the enzyme captured by the antibodies was measured by adding H_2_O_2_ (10 μM), nitrite anions (NO_2_^−^, 10 mM) and Amplex^®^ Red (40 μM) as fluorogenic substrate. The oxidation of Amplex^®^ Red into the fluorescent adduct resorufin (*λ*_excitation_ = 544 nm; *λ*_emission_ = 590 nm) was monitored for 30 min at 37 °C with a fluorescent plate reader (Fluoroskan Ascent, Fisher Scientific). A control assay set as 100% MPO activity was performed with purified MPO in the presence of PBS instead of the plant extracts.

### 3.7. Phytochemical Analysis

#### 3.7.1. Extract Preparation

Plant powder (1.5 g) was stirred in 250 mL ultra pure water and simultaneously heated 30 min at 60 °C under backward flow until filtration on a Whatmann paper filter (125 mm). 50 mL supernatant were removed before the filtrate was diluted with ultra pure water (1/4 v/v), giving the start sample that was used in the following determinations.

#### 3.7.2. Determination of Total Polyphenol in the Extracts

Total polyphenol was determined with Folin-Ciocalteu reagent according to the method described by Singleton *et al.* (1999) [65]. An aliquot of 200 μL of the sample prepared above was mixed with 100 μL Folin-Ciocalteu reagent in 1 mL ultra pure water and vortexed. 1.2 mL of an aqueous solution of Na_2_CO_3_ (200 g/L) were added and the mixture was stored for 30 min at room temperature in darkness before the absorbance was measured at 760 nm on an UVIKON 922 spectrometer using H_2_O as blank.

#### 3.7.3. Determination of Tannins in the Extracts

To determine the tannin content, 100 mg sheepskin powder were dissolved in 10 mL start sample prepared above (3.7.1) and magnetically shaken for 1 h at room temperature. The volume of the resulting filtrate was increased with ultra pure water to 1:4 v/v, giving a solution whose aliquot (200 μL) was treated on the same way as for the determination of total polyphenol (3.7.2).

The absorbance was read at 760 nm, using pyrogallol as control and water as blank (Spectophotometer UVIKON 922). The results, expressed as g pyrogallol equivalent/100 g dry plant powder, were calculated according to the formulae: total polyphenol = [(62.5 × *A*_1_) × *m*_2_]/(*A*_3_ × *m*_2_), not tannin polyphenol = [(62.5 × *A*_2_) × *m*_2_]/(*A*_3_ × *m*_1_) and tannins = [62.5 × (*A*_1_ − *A*_2_) × *m*_2_]/(*A*_3_ × *m*_1_), whereby *A*_1_, *A*_2_ and *A*_3_ are the absorbance values of total polyphenol (*A*_1_), not tannin polyphenol (*A*_2_) and pyrogallol (*A*_3_), *m*_1_ and *m*_2_ are the mass (in gram) of respectively the plant powder (*m*_1_) and pyrogallol (*m*_2_) that were weighed for this determination.

#### 3.7.4. HPLC-UV/DAD Determination of Total Flavonoid and Phenol Acid

The samples were extracted from 1 g plant powder boiled for 5 min in 10 mL methanol at 60 °C and filtered on hydrophilic cotton. 1 mg of respectively rutin, hyperoside, caffeic acid, chlorogenic acid and rosmarinic acid of HPLC analytical grade was dissolved in 10 mL methanol and gently shaken. The total flavonoid and phenol acid was determined at 25 °C by an “Agilent 1100” HPLC chain connected to a diode array detector (DAD). All samples were filtered through a 0.45 μm pore size syringe-driven filter before their injection (20 μL) into the HPLC-UV/DAD system. The separation was carried out using an Hypersil ODS column (4 mm × 250 mm) with a nonlinear gradient of acetonitril (solvent A) and 0.05% trifluoroacetic acid in ultra pure water (solvent B) in the following composition: T_0_: 0% A, 100% B; T_1_: 3% A, 97% B; T_45_: 40% A, 60% B; T_46_: 0% A, 100% B and T_60_ stop, the time (T) is expressed in minutes. The compounds were eluted at a flow rate of 1 mL/min and detected with UV-DAD. The UV spectra corresponding to the elution peaks were recorded in the range from 250 to 340 nm and the chromatograms were monitored at 280 and 340 nm. Calibration curves were calculated with increasing concentrations of hyperoside and chlorogenic acid to express the total flavonoid and phenol acid contained as hyperoside (for flavonoids) and chlorogenic acid (for phenolic acids) weight equivalent in 100 g of dry plant powder.

### 3.8. Statistical Analysis

Within an experiment, each measure was repeated three times, and each assay was done at least three times (*n* ≥ 9). The statistical analysis was performed with GraphPad Instat 3.05 (GraphPad Software, San Diego CA, USA). A One-way Analysis of variance (ANOVA) and a “Student-Newman- Keuls Multiple Comparisons” test were carried out. The IC_50_ and R^2^ values were calculated with GraphPad Prism 5.0 after converting the concentrations into their decimal logarithm and applying the function “log (inhibitor) *vs*. normalized response-variable slope”. All results are expressed as mean ± standard deviation (SD) in percentage *vs.* controls, which were set as 100%. * *p*-value < 0.05 was considered as significant.

## 4. Conclusion

Our findings provide new insights into the AOX, anti-radical, anti-inflammatory, and modulating properties of *Abelmoschus esculentus*, *Hibiscus acetosella*, *Manihot esculenta* and *Pteridium aquilinum* in “inflammation like” conditions. The dietary plants that we have tested were shown to contain polyphenols and flavonoids with well-known AOX activities, as previously reported by our team [35]. Using neutrophils as cellular model, we now evidence that molecules of these plant extracts not only act stoichiometrically as radical scavengers, but also anti-catalytically towards key-enzymes involved in the inflammatory response of neutrophils, especially MPO. The data evidence the importance to achieve deeper views into the profile of the AOX compounds contained in these plants, because they can be a useful source for an efficient AOX dietary intake and eventually of therapeutic interest in the regulation of chronic or acute inflammatory diseases.

## Figures and Tables

**Table 1:** IC_50_ (μg/mL) and R^2^ (between brackets) values of not heated and boiled plant extracts on ROS production (CL assay), tyrosine nitration activity and nitration-peroxidasic activity (SIEFED) of MPO, expressed as mean ± SD (*n* = 9). Abel = *Abelmoschus*; Hib = *Hibiscus*; Man = *Manihot*; Pter = *Pteridium*

	IC_50_ (μg/mL)					
	Chemiluminescence		Tyrosine nitration		SIEFED technique	
	not heated	boiled	not heated	boiled	not heated	boiled
Abel	10.40 ± 1.1 (0.901)	10.77 ± 1.0 (0.942)	10.06 ± 1.0 (0.962)	10.92 ± 1.6 (0.913)	13.57 ± 1.1 (0.834)	14.71 ± 1.1 (0.824)
Hib	1.70 ± 0.8 (0.890)	2.23 ± 1.1 (0.897)	8.77 ± 1.1 (0.851)	9.81 ± 1.1 (0.889)	28.72 ± 1.2 (0.806)	30.55 ± 1.6 (0.579)
Man	4.82 ± 1.0 (0.965)	5.04 ± 1.0 (0.970)	4.92 ± 1.0 (0.963)	5.23 ± 1.0 (0.966)	4.96 ± 1.0 (0.967)	5.04 ± 1.0 (0.996)
Pter	1.43 ± 0.8 (0.907)	1.89 ± 0.8 (0.688)	1.10 ± 0.7 (0.827	1.14 ± 0.4 (0.940)	1.50 ± 0.8 (0.917)	1.28 ± 0.7 (0.921)

**Table 2:** Quantitative determination of flavonoid, phenolic acid, total polyphenol and tannin content (mg/100 g dry weight) of the plant powders calculated by HPLC-UV/DAD analysis and with Folin-Ciocalteu reagent.

	*Abelmoschus*	*Hibiscus*	*Manihot*	*Pteridium*
Total polyphenol a	1,480	1,730	N.D.	6,740
Tannin b	600	500	N.D.	5,550
Non-tannin	880	1,230	N.D.	1,190
Total Flavonoid c	425	775	1,381	448
Total phenolic acid d	N.D.	975	N.D.	278

a Total polyphenol was calculated by the Folin-Ciocalteu method using pyrogallol as reference, and is expressed as pyrogallol weight-equivalent in 100 g of dry plant powder;

b Tannins were determined by reaction with sheepskin powder followed by Folin-Ciocalteu method and expressed as pyrogallol weight-equivalent in 100 g of dry plant powder;

c Total flavonoid was determined by HPLC-UV/DAD analysis by integrating the total surface under the elution peaks of flavonoids found on a calibration curve calculated with hyperoside at increasing concentrations, and is expressed as hyperoside weight-equivalent in 100 g of dry plant powder;

d Total phenolic acid was determined by HPLC-UV/DAD analysis by integrating the total surface under the elution peaks of the phenolic acids found on a calibration curve calculated with chlorogenic acid at increasing concentrations, and is expressed as weight-equivalent of chlorogenic acid in 100 g of dry plant powder. N.D.: not determined.
